# Streamflow Impacts of Biofuel Policy-Driven Landscape Change

**DOI:** 10.1371/journal.pone.0109129

**Published:** 2014-10-07

**Authors:** Sami Khanal, Robert P. Anex, Christopher J. Anderson, Daryl E. Herzmann

**Affiliations:** 1 School of Environment and Natural Resources, Ohio State University, Wooster, OH, United States of America; 2 Dept. of Biological Systems Engineering, University of Wisconsin-Madison, Madison, Wisconsin, United States of America; 3 Dept. of Agronomy, Iowa State University, Ames, Iowa, United States of America; DOE Pacific Northwest National Laboratory, United States of America

## Abstract

Likely changes in precipitation (P) and potential evapotranspiration (PET) resulting from policy-driven expansion of bioenergy crops in the United States are shown to create significant changes in streamflow volumes and increase water stress in the High Plains. Regional climate simulations for current and biofuel cropping system scenarios are evaluated using the same atmospheric forcing data over the period 1979–2004 using the Weather Research Forecast (WRF) model coupled to the NOAH land surface model. PET is projected to increase under the biofuel crop production scenario. The magnitude of the mean annual increase in PET is larger than the inter-annual variability of change in PET, indicating that PET increase is a forced response to the biofuel cropping system land use. Across the conterminous U.S., the change in mean streamflow volume under the biofuel scenario is estimated to range from negative 56% to positive 20% relative to a business-as-usual baseline scenario. In Kansas and Oklahoma, annual streamflow volume is reduced by an average of 20%, and this reduction in streamflow volume is due primarily to increased PET. Predicted increase in mean annual P under the biofuel crop production scenario is lower than its inter-annual variability, indicating that additional simulations would be necessary to determine conclusively whether predicted change in P is a response to biofuel crop production. Although estimated changes in streamflow volume include the influence of P change, sensitivity results show that PET change is the significantly dominant factor causing streamflow change. Higher PET and lower streamflow due to biofuel feedstock production are likely to increase water stress in the High Plains. When pursuing sustainable biofuels policy, decision-makers should consider the impacts of feedstock production on water scarcity.

## Introduction

As demand for renewable fuels grows, biofuels from lignocellulosic feedstock are considered a promising alternative to corn-based ethanol [1]–[2]. Cellulosic biofuels are expected to be both environmentally and energetically superior to grain-based biofuels [3]–[6]. The mandate set by the Renewable Fuel Standard [7] to use 16 billion gallons of cellulosic biofuel per year by 2022 is projected to have significant impact on agricultural land use in the U.S. as lands are converted for the production of bioenergy crops [8]. Prior studies [6], [9] have investigated yields, land use, economics and greenhouse gas emissions of bioenergy crops, but one key factor often overlooked is the hydrologic balance associated with bioenergy crop production.

There is strong coupling between the land surface and atmosphere that is heavily influenced by the vegetative land cover [10]–[13]. Change in land cover thus has the potential to impact local and regional climate through alteration of the energy and moisture balances of the land surface [14]–. The longer growing season and greener vegetative cover of biofuel crops result in higher water loss to the atmosphere through evapotranspiration (ET), decline in soil water depth [17], [19] and reduced surface runoff [20] relative to annual cropping systems. Changes in soil moisture and runoff determine streamflow, groundwater recharge and influence water quality.

Bioenergy crops, e.g., switchgrass and miscanthus, can transpire as much as 38% more than corn over a growing season [20]. Replacing traditional annual cropping systems with switchgrass in the Midwest and High Plains may cause additional stress to water resources because the agricultural crop production in large portions of these areas (e.g., Kansas and Nebraska) is dependent upon irrigation water from already stressed local resources [21]. Streamflow volume (Q) is responsive to changes in both climate and land cover [22]–[23], and changes in Q have important biological and socioeconomic implications [24]–[26]. Anthropogenic alteration of Q has been shown to impair aquatic communities and ecosystems, and the likelihood of impairment rises rapidly with increasing severity of reduced Q [25].

Water may be a significant limiting factor for biofuel crop production in many agricultural regions. It is important that we develop projections of future water use for agricultural crop production under climate change induced by land use change and to account for the impact of that water use on critical water resources. Prior studies have examined the potential for biofuel crops to affect regional climate [15]. The climate feedback of biofuel crops examined in these studies, however, is based on hypothetical scenarios that do not account for the socio-economic responses of land managers and thus do not represent plausible land use patterns that might result from current biofuel policies. To our knowledge, no prior studies have explored the changes in streamflow volume in response to climate change induced by land use/land cover (LULC) change; certainly none have examined this under the constraints of enacted legislation. Future projections of climate and climate-driven streamflow under plausible landscape scenarios will aid state and federal agencies in assessing the local cost of adaptation, increase public awareness, and guide the development of new mitigation programs related to water resources.

In this study, we examine changes in hydrologic processes including precipitation (P), ET, PET, runoff and Q that result from modification of local/regional climate driven by switchgrass cropping systems predicted to replace current cropping systems in the High Plains (hereafter referred to as the “biofuel scenario”). A regional climate model coupled to a land surface model is used to capture feedback between changes in the vegetation canopy due to switchgrass planting and regional climate processes. The change in Q to climate change under the biofuel scenario compared to the current cropping system scenario (hereafter referred to as the “baseline scenario”) is estimated based on widely used non-parametric approaches [22]–[23], [27]. These non-parametric approaches utilize the concept of elasticity of Q that is usually derived using the historic relationship between Q, P and PET. Following the similar approach, we first derived the elasticity of Q to climate, and later used it in combination with projected changes in P and PET to derive changes in Q under the biofuel scenario relative to baseline across the conterminous U.S.

## Materials and Methods

### Regional climate modeling framework

**Figure 1:**
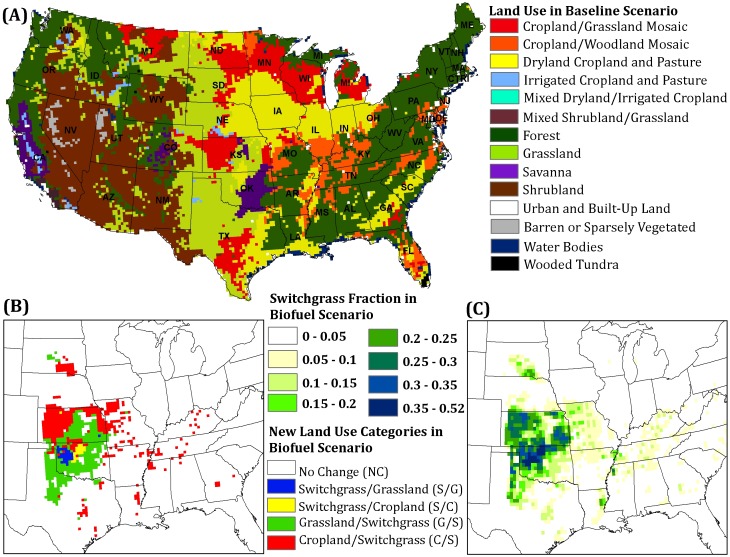
Default land use categories in the WRF model (A); new land use categories defined for the biofuel scenario (B); and fraction of land use that is switchgrass in the biofuel scenario (C).

The Weather Research Forecast (WRF) model version 3.1.1 [28]–[29] and NOAH land surface model (NOAH LSM) are used for regional climate simulations. The simulation domain covers the continental U.S. at a resolution of 0.25 degree (i.e., 24 km). The NOAH LSM coupled to the WRF model is used to represent the interaction of soil and vegetation with the atmosphere [30]. Regional climate simulations were produced for the period of 1979–2004 under the two land use scenarios. The choice of the simulation period is constrained by 1) availability of data describing the baseline scenario; 2) the accuracy of extrapolating land use categories derived from 1991–1995 satellite data further into the future; and 3) the large computational burden associated with longer simulations. The baseline scenario represents land use categories and monthly phenology based on satellite derived data from 1991–1995, and the biofuel scenario represents projected alternative (i.e., switchgrass) land use categories (Figure 1) with identical atmospheric forcing data. Following the recent studies [16], [31]–[32] that have used 2-years as a minimum length for spin-up, we discarded the first two years (i.e., 1979 and 1980) of each simulation to allow for adjustment of the land surface with the atmosphere. Details of the model configuration are provided in the Anderson et al [19], and thus are not described here.

### Land use scenarios

The baseline scenario uses the NOAH LSM default settings of land use and vegetation parameters, including 24 vegetation classes, a vegetation parameter table and satellite-based (1991–1995) monthly vegetation fraction from which leaf area index (LAI) and albedo are derived (Table S1). The projection of LULC change produced by the Policy Analysis System (POLYSYS) model [33]–[34] in support of the DOE study report “U.S. Billion-Ton Update” [7] is used to create the biofuel land use scenario. The Billion Ton Update study examines the feasibility of attaining annual production of one billion dry tons of biomass feedstock by 2030 based on projections of future biomass demand, inventory, production capacity, availability, and technology. In the 2011 update, the greatest conversion of traditional cropping system to switchgrass occurs in the Great Plains, with 20–30% in Kansas and 30–45% in northern and northwest Oklahoma [35]. The POLYSYS simulation contained county level switchgrass and crop production for 2022, the first year Renewable Fuel Standard (RFS) goals reach maximum levels, at a $60/dry ton farm gate switchgrass price with the Billion-Ton Study baseline assumptions, including an extension of the USDA 10-year yield forecast for major food and forage crops to 2022. An area weighted method was used to resample county-level POLYSIS estimates of switchgrass conversion to the WRF grid [19]. The NOAH LSM uses a single vegetation category for each grid cell, and vegetation parameters are homogenous within each grid cell. Thus, a grid cell that contains a mixture of vegetation types does not explicitly account for each type, but represents an average of vegetation parameters over all vegetation types present in the grid cell.

For the biofuel scenario, four new vegetation classes, their related vegetation parameters and monthly vegetation fraction are introduced in the WRF model based on default land use categories (Figure 1A). The new vegetation classes are modified to represent a mixture of the default land use categories and switchgrass (Figure 1B). This reflects that a mixture of biofuel and conventional crops are expected in regions where biofuel crops are adopted rather than complete replacement of conventional crops with switchgrass. Two of the new classes (Switchgrass/Grassland Mosaic and Switchgrass/Cropland Mosaic) are used for grid cells in which the switchgrass fraction exceeds 30%, and two (Grassland/Switchgrass Mosaic and Cropland/Switchgrass Mosaic) are used for grid cells in which the switchgrass fraction is below 30% [36] (Figures 1B and 1C). We prevented land use change to switchgrass in regions beyond that projected by the Billion ton update by only changing the parameters based on latitude/latitude. For example, land cover changes were mostly in Oklahoma and Kansas (Figure 1B); we did not change parameters in California.

As the new land use categories under the biofuel scenario reflect a mixture of switchgrass and conventional crops rather than complete replacement of switchgrass, the phenology for new land use categories is characterized in our simulations by adjusting the satellite-based monthly vegetation fraction based on discussion with scientists working on field trials of switchgrass. The monthly greenness fraction is the basis for LAI and albedo calculations in NOAH LSM, and both LAI and albedo increase with vegetation fraction. During the growing season, spectrally weighted albedo (which is required in WRF) increases as a crop is greening up. To represent change in phenology consistent with managed plots of switchgrass stands, vegetation fraction is increased during February–October to simulate earlier greening, denser foliage at peak LAI, and later senesce of switchgrass (a perennial grass) compared to annual crops and rangeland. Increase of vegetation fraction ranges 10–20%, except north central Oklahoma where it is increased by 90% to offset low LAI due to winter wheat harvest [19].

The maximum and minimum values of LAI and albedo are adjusted to reflect changes in vegetation under the biofuel scenario (Table S1). And, the same monthly phenology is imposed for each simulated year. When the vegetation fraction is at its peak, the LAI and albedo are as well. Maximum LAI is based upon observations of field stands of managed perennial grass that grows a denser canopy than prairie grass. Although LAI>6 as measured in field trials of managed switchgrass is used in simulations of switchgrass production [37]–[38], maximum LAI<6 is set to reflect a regional vegetation mixture. This approach is consistent in simulations with Van Loocke *et al* [17].

### Climate elasticity of streamflow and changes in streamflow

In this version of WRF model, surface runoff is computed as the excess of precipitation that does not infiltrate into the soil [39]. Although NOAH-LSM describes the canopy and root zone in detail, the interactions between groundwater, the root zone, and surface water were not yet included at the time our project was undertaken and completed. This version parameterizes surface runoff with a simple infiltration-excess scheme rather than terrain slope channel routing, and it treats baseflow as a linear function of bottom soil-layer drainage [40]. Thus, runoff estimates from WRF are not representative of the changes in streamflow. For the purpose, we used non-parametric approaches that are demonstrated as or more robust than complex and detailed hydrologic models for evaluating the sensitivity of streamflow to climate [22], [27], [41]. These non-parametric approaches use the concept of climate elasticity of streamflow (ε_x_), computed based on historic P, PET and Q data.The climate elasticity of streamflow is defined by the proportional change in Q to the change in a climate variable (x), such as P or PET [42]. It is an index commonly used to quantify the sensitivity of Q to changes in climate. Often this index (i.e., ε_x_) is derived from the historic climate and hydrologic data (i.e., P, PET and Q) [23], [42]. Streamflow in unimpaired watersheds (i.e., watersheds in which streamflow are not subject to regulation or diversion, and defined as reference watershed in this study) can be modeled as a function of P and PET [23]. The changes in Q due to changes in P and PET can be approximated as:
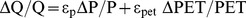
(1)


In equation 1, ΔQ, ΔP and ΔPET are changes in Q, P and PET, respectively; ε_p_ and ε_pet_ are the elasticities of streamflow with respect to P and PET. Prior studies [23], [27], [43] have proposed non-parametric approaches to estimate ε_x_ from observed climatic data. Of the various approaches (Table S2, Figure S2) found in the literature, we have no reason to favor one over the others, and thus use the average of ε_p_ estimated from all available non-parametric approaches to predict average changes in Q under the alternative LULC scenario. Details about the non-parametric approaches used in this study and hydro-climatology of the conterminous U.S are discussed in Figure S3.

To compute elasticity estimates, we used historical annual streamflow, precipitation and PET information for 1,845 reference watersheds across the conterminous United States (see Figure S1). To compute ε_x_ estimates from the historic climate record, we used PET instead of ET similar to prior studies [22]–[23] due to data limitations associated with the estimation of actual ET from 1950–2009. Further, as ε_pet_ was computed using historic PET estimates, we used PET instead of ET from the regional climate model simulations to estimate changes in Q to maintain consistency in the methodology. These climate elasticity values were combined with the differences in mean annual P and PET between the biofuel and baseline scenarios, expressed as a percentage of the baseline, to compute the relative change in Q across the nation under the biofuel scenario (equation 1).

### Sensitivity of streamflow change

Uncertainty in the estimated change in Q under the biofuel scenario is evaluated based on: 1) difference in elasticity estimates computed from various non-parametric approaches (discussed in the supporting material), and 2) year to year changes in simulated P and PET for the period 1981–2004. To evaluate the sensitivity of changes in streamflow to changes in both climate and elasticity estimates, we calculated the standard deviation (std) of percent annual change in P and PET between the biofuel and baseline scenarios, and the std of elasticity estimates from the seven different non-parametric methods as shown in equations 2 and 3.
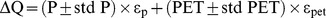
(2)

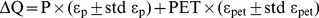
(3)

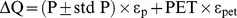
(4)


In equations 2, 3 and 4, P and PET indicate the percent change in precipitation and evapotranspiration under the biofuel scenario relative to the baseline. ε_p_ and ε_pet_ indicate the mean values of ε_p_ and ε_pet_ from the seven empirical methods.

The sensitivity of estimated change in Q is evaluated by varying the percent change in mean annual P and PET under the biofuel scenario (equation 2; Figure S6A and S6B), and the mean estimates of ε_pet_ and ε_p_ (equation 3; Figures S6C and S6D) by ± their standard deviation. We estimated the sensitivity of predicted change in Q by varying the percent change in mean annual P and PET simultaneously because P observed in many regions including High Plains are correlated with PET [44] and ET [45]. Sensitivity measures are also computed varying only P (equation 4; Figure S7) because the impacts of varying P and PET simultaneously will tend to cancel each other in the regions where impacts are inversely correlated, thus not reflecting the full contribution of P or PET to Q (equation 4).

## Results

### Climate elasticity of streamflow

**Figure 2:**
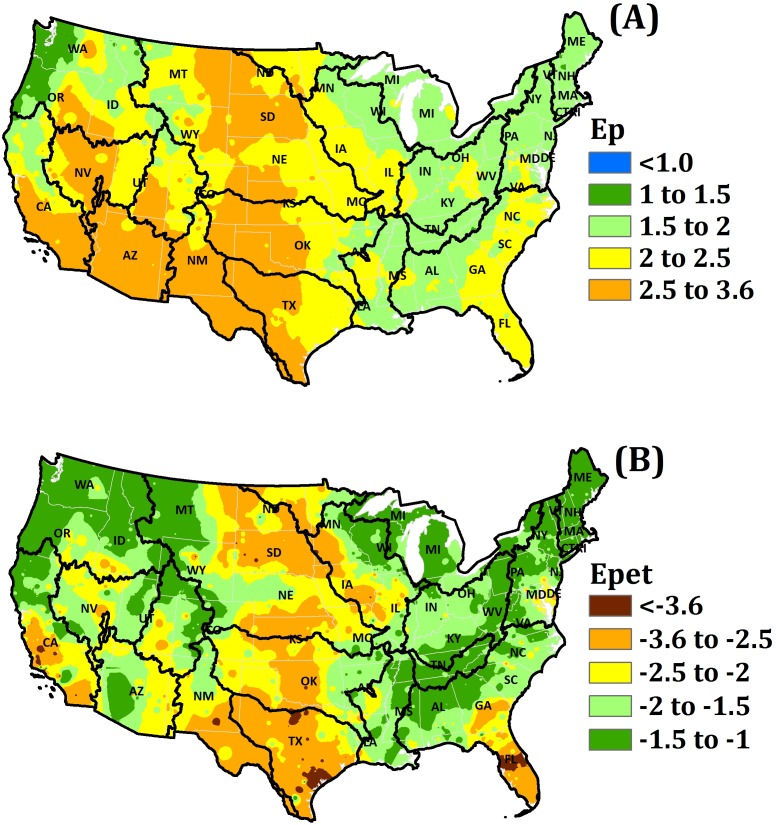
Hydro-climatology of the conterminous US; (A) Precipitation elasticity of streamflow (ε_p_) and (B) Evapotranspiration elasticity of streamflow (ε_pet_).

Precipitation elasticity of streamflow is estimated in the range of 1–3.6 with a mean of 2.2 for watersheds across the U.S., implying that a 1% change in P will result in more than a 1% change in Q. The relationship between P and Q is generally non-linear, and this non-linearity is influenced by catchment properties including storage processes, ET and vegetation properties, and these factors are implicitly factored into elasticity estimates. Approximately 46% of all watersheds examined have ε_p_ higher than 2, and these watersheds are clustered in the Southwest, Midwest and Southeastern parts of the nation (Figure 2A) where PET usually exceeds P. Only a few basins in the Northwest have ε_p_ less than 1.5.

Evapotranspiration elasticity of streamflow for watersheds across the U.S. is estimated to be in the range of negative 3.6 to negative 1, with a mean of negative 1.9, indicating that a 1% reduction in PET would result in about a 1.9% increase in Q. The geographical distribution of ε_pet_ (Figure 2B) in the conterminous U.S. is similar to the distribution of ε_p_ with lower values ε_pet_ in the arid and semiarid regions of the Southwest and Midwest.

### Variability in climate elasticity of streamflow

The variability in elasticity estimates computed using seven different non-parametric approaches (expressed as a standard deviation) is high in the arid and semi-arid regions of the Midwest and Southwest, and lower in the humid and semi-humid regions (Figures S4A and S4B). Also, the standard deviation of ε_pet_ estimates is higher than the standard deviation of ε_p_ estimates. This is due to differences among the seven different approaches; five of seven approaches [46]–[51] depend upon aridity index (PET/P) to estimate elasticity estimates, while the other two depend on Q and P or PET [23], [27] (see Table S2 for details).

### Projected climate change under biofuel scenario

#### Change in annual precipitation and evapotranspiration

**Figure 3:**
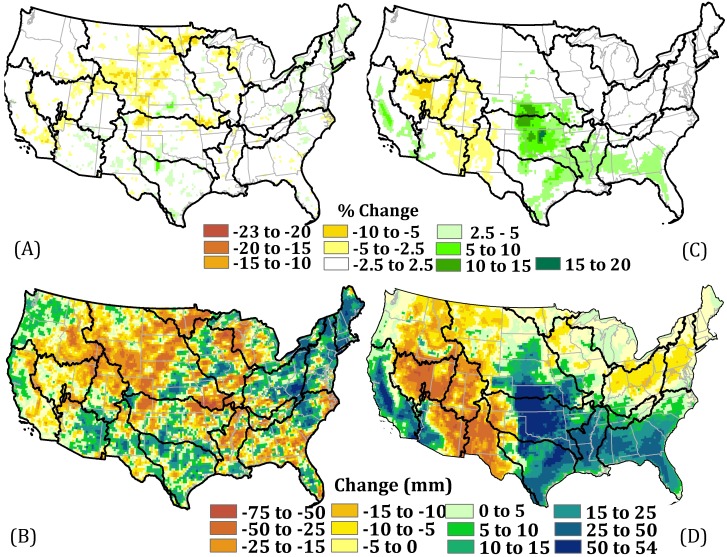
Change in mean annual precipitation and potential evapotranspiration for 1981–2004 expressed in A) percentage change in mean annual precipitation; B) change in mean annual precipitation (millimeters); C) percentage change in mean annual PET; and D) change in PET (millimeters) under the biofuel scenario. Percent change under the biofuel scenario relative to the baseline scenario for a given location is estimated as the mean of: 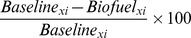 where x is either P or PET and i is year between 1981 and 2004.

Under the biofuel scenario, the mean annual change in P relative to the baseline is projected to be in the range of negative 10% to positive 10% (Figure 3A). About 84% of the conterminous U.S. is predicted to experience changes in P in the range of negative 5% to positive 5% under the biofuel scenario; and a general decrease in P is predicted over 52% of the area. The magnitude of projected change in mean annual P in the main biofuel crop producing region (i.e., Kansas and Oklahoma) is between 2.5% to 5% relative to the baseline scenario. Under the biofuel scenario, western Kansas and Oklahoma show 5 to 15 mm higher annual P than under the baseline scenario.

The projected change in mean annual PET under the biofuel scenario is estimated to be in the range of negative 6% to positive 16% relative to the baseline (Figure 3C). Higher PET in the switchgrass planted region is consistent with lower mean temperatures due to earlier green-up and the higher LAI of switchgrass compared to current vegetation, and higher net radiation under the biofuel scenario.

In addition to the observed changes in climate in switchgrass planted region, we observed changes in climate patterns in areas away from the switchgrass concentrated area. Under the biofuel scenario, southern regions including parts of Arizona, New Mexico and Texas, the High Plains including western Kansas and Oklahoma, the Midwest including eastern part of Nebraska and Iowa, and a large region of the eastern states show an increase in annual P of between 2.5% to 10% relative to the baseline scenario. A large decline in annual P (i.e., between 2.5% to 10% relative to the baseline) is predicted across much of the northern (i.e., northern Minnesota, South Dakota, Wisconsin), Midwestern (i.e., Wyoming, Idaho, northern Colorado) and the High Plains (i.e., Missouri) regions. Due to the internal non-linear climate dynamics, a single simulation is insufficient to conclude that they are systematically caused by land use change in the Great Plaines. They could, in fact, be an artifact of the initial atmospheric conditions.

**Figure 4:**
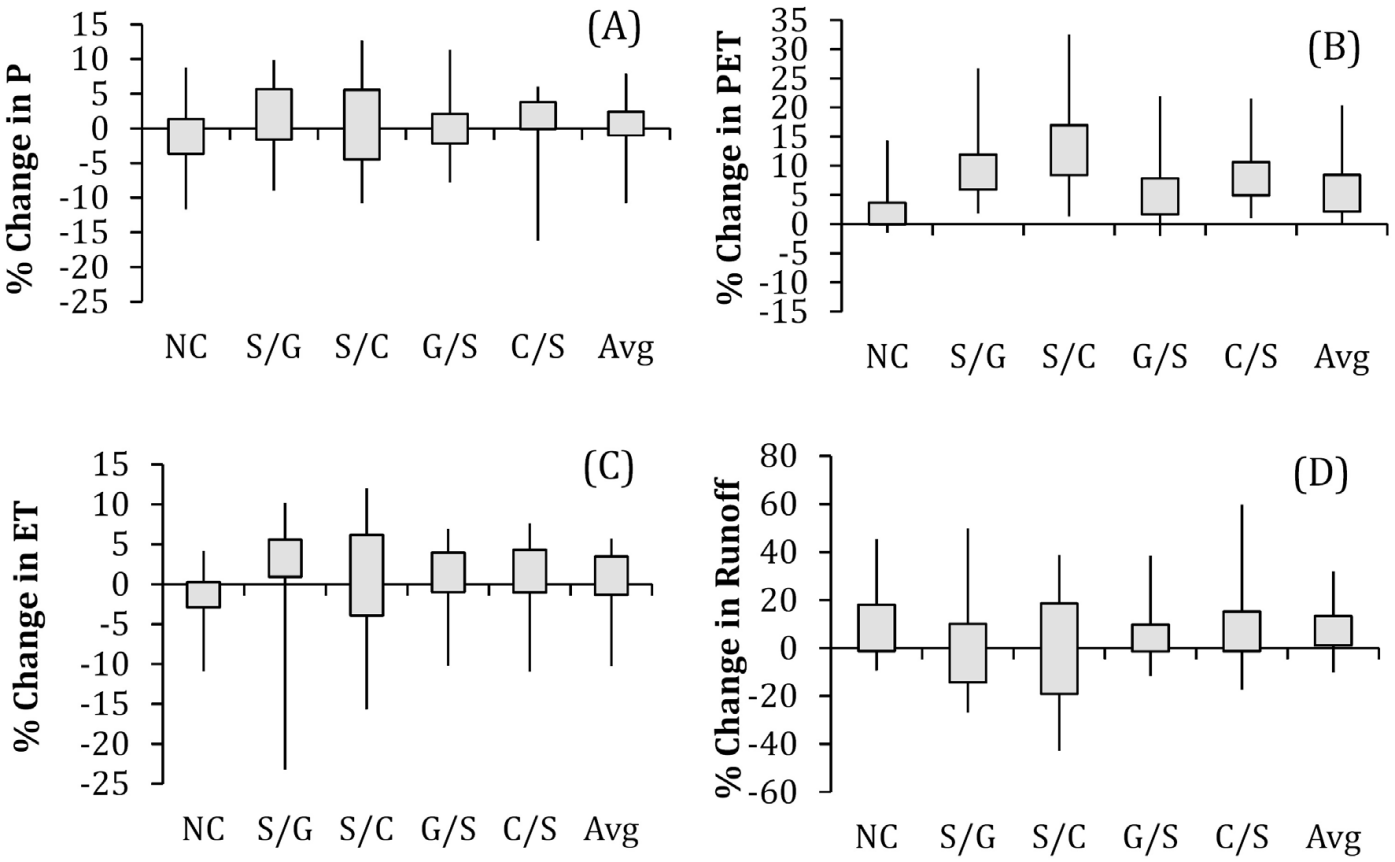
Percent difference in (A) annual precipitation; (B) PET averaged by land use categories in switchgrass altered regions in Kansas and Oklahoma as shown in **Figure 1**. Box top and bottom edges are the interquartile range of percent difference for each year, and whiskers are maximum and minimum annual values. X-axis labels are land use categories: No Change (NC), Switchgrass/Grassland (S/G), Switchgrass/Cropland (S/C), Grassland/Switchgrass (G/S), Cropland/Switchgrass (C/S), and average over all categories (Avg).

PET is usually a good representation of actual ET when there is no plant water stress, and is thus commonly used in precipitation-runoff modeling applications [41]. We observed similar trends in PET and ET in the switchgrass perturbed region (Figures 4B and 4C) although they differed in magnitude. Increases in ET increase low-level humidity and the potential for more P [19]. Change in mean annual P, PET, ET and runoff when examined by land use (i.e., switchgrass altered and unaltered) categories in the High Plains, suggests that the difference in ET, PET and runoff represent a change induced by land cover perturbation (Figures 4 and S5).

Differences in climate between the baseline and biofuel projections vary annually in both magnitude and direction between 1981 and 2004. The magnitude of mean annual change in PET and ET is higher than the year to year changes in PET and ET in switchgrass planted regions under the biofuel scenario. Also, the mean annual runoff in the switchgrass dominated region is lower than other regions in Kansas and Oklahoma. Compared to land cover with a lower fraction of switchgrass (i.e., cropland/switchgrass and grassland/switchgrass), the land cover with a large fraction (i.e., >30%) of switchgrass (i.e., switchgrass/grassland and switchgrass/cropland) demonstrated a higher magnitude of change in PET, ET and runoff in the biofuel scenario (Figure 4). This indicates that the observed changes in PET, ET and runoff associated with biofuel feedstock production are large and significant. Decrease in runoff results from lower soil moisture levels due to higher evapotranspiration of switchgrass during the growing season [19]. Conversely, the inter-annual variability of change in P is as large as or larger than the magnitude of mean annual change in P (Figure 4). Thus it is hard to conclude that P is changed under the biofuel scenario.

#### Streamflow response to projected climate change

**Figure 5:**
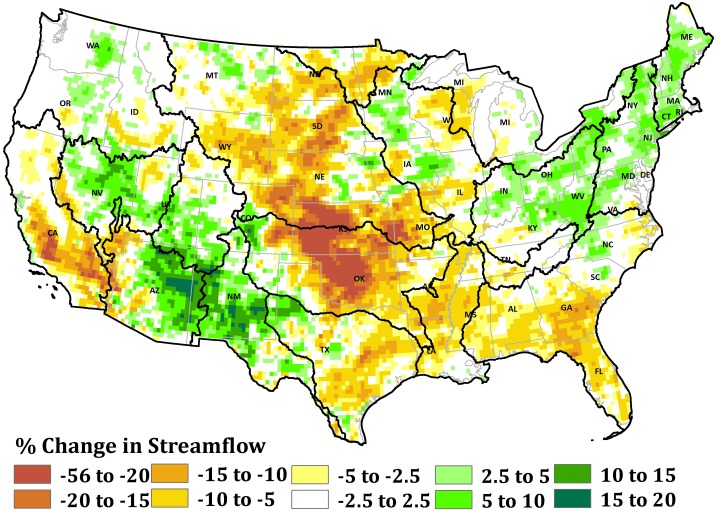
Percent change in mean annual streamflow as a function of change in annual precipitation and potential evapotranspiration under the biofuel scenario.

Mean annual change in Q in response to changes in P and PET under the biofuel scenario is shown in Figure 5. Across the conterminous U.S., the change in mean Q under the biofuel scenario is estimated to be in the range of negative 56% to positive 20% relative to the baseline scenario. An increase in Q with magnitude greater than 5% is predicted over 12% of the area. Lower PET but higher P in New Mexico and Arizona are estimated to increase Q under the biofuel scenario. However, a decrease in Q with magnitude greater than 5% is predicted over 30% of the area. The increase in P is smaller than the increase in PET under the biofuel scenario, and this causes a net decline in Q in the High Plains. In the High Plains, Q is predicted to be about 20% lower than the baseline. Streamflow in the biofuel crop region within the High Plains is 18% lower relative to the baseline (Figure 5). The switchgrass areas in the biofuel crop region show decreases in streamflow that are twice as large as the decrease in the unperturbed area.

#### Sensitivity of streamflow

The change in Q under the biofuel scenario is observed to be highly sensitive to mean annual P and PET for large parts of the conterminous U.S., however, it is less sensitive in the switchgrass planted region where change in PET is larger than P (Figure S6). As parameters associated with precipitation and evapotranspiration are very likely to be correlated with each other, PET and P were changed independently in the sensitivity test to examine the relative significance of changes in P and PET on streamflow estimates (see the section ‘Materials and Method’ for details). Under these sensitivity analyses, Q always decreases under the biofuel scenario, even in the case when PET is held constant and P is increased by one standard deviation (Figure S7). Sensitivity analysis also showed that predicted changes in streamflow are robust to differences in ε_p_ and ε_pet_ stimation approaches. Change in Q across the range of ε_p_ and ε_pet_ estimation approach (Figure S6C and S6D) is less than the change in Q resulting from changes in P and PET equal to their inter-annual variability (Figure S6A and S6B).

## Discussion

Climate change is a likely result of a policy that encourages expansion of energy crop production in the High Plains and Midwest U.S [19]. This climate change is caused by alteration of the surface energy and moisture balances induced by changes in land cover when current cropping systems are replaced by energy crops. Notable changes include lower temperature, higher PET, lower runoff and a decline in Q in the region where switchgrass is predicted to replace current vegetation. Our analyses show changes in the mean annual PET, ET and streamflow to be a stronger climate change impact of biofuel crop production than changes in mean annual P.

The main goal of this study is to use a simple and robust model to communicate to policy makers and analysts the potential implications of biofuels policy-induced climate change on streamflow to inform the search for a sustainable renewable fuel production system. While adopting the simple but robust model for estimating streamflow changes, we made several assumptions, and these assumptions are likely to introduce uncertainties in our streamflow estimates. Despite some of the limitations (as discussed below), our conclusion that increase cellulosic feedstock production are likely to reduce water yield is found to be in agreement with other studies conducted [52], [20] in other parts of the nation.

Weather conditions simulated with regional and global climate models over short periods are sensitive to their initial conditions. A change in the vegetation type at the initial condition will result in a different sequence of weather conditions. Thus, differences between simulations may be a combination of a transient response from unpredictable nonlinear dynamics acting upon a different initial state as well as a systematic response from a structural change in forcing of local climate, in this case land use change [53]–[54]. The average change for a transient response is expected to be zero given either a long data record or multiple simulations from alternative initial conditions. Changes in Q estimated in this study thus include uncertainties inherent in P and PET estimated over a relatively short time series (in this case 24 years). Anderson et al [19], using one-way ANOVA analysis, found statistically significant change of monthly values for ET and P. However, the small change in annual ET resulted from offsetting statistically significant monthly changes. To examine further whether the ET and P responses were possibly transient, they performed a second simulation of a single year of the control design beginning from a different initial condition. The difference of ET in the two control simulations was much smaller than the difference between the control and LULC scenario. Precipitation, however, showed substantial sensitivity to the initial condition. This sensitivity of P to initial conditions indicates a larger number of simulations would be needed to identify a forced response in P, if one exists at all. Therefore, we are unable to state that a forced change in P exists anywhere in our simulation domain. Since P, ET and PET are correlated, we conclude that outside of the switchgrass planted region, change in PET and Q should not be considered a forced response. Within the switchgrass planted region, however, change of PET and Q is a forced response. Another source of uncertainty in our estimates can be driven by the use of temperature based approach to estimate PET instead of approach like Penman-Monteith (see the supporting material). As temperature-based PET estimates are likely to overestimate the impacts of changes in temperature, our streamflow estimates might have been overestimated to some extent.

Another possible area of limitation includes elasticity estimates. In the study, we interpolated the elasticity estimates based on reference watershed for the conterminous US. This means, our elasticity estimates may not accurately reflect the connection of climate to Q in non-reference watersheds where streamflow is heavily influenced by land use practices and ground water pumping. Predicted change in Q in non-reference watersheds thus might be over or under estimated in the biofuel scenario. Despite this limitation, this study is useful in providing the direction of change in streamflow under the biofuel scenario without requiring the use of detailed hydrologic models that are computationally complex and often provide ambiguous results when compared [27].In the study we show a potential change in annual streamflow volume as an outcome of a landscape influenced climatic system. Our analyses also suggest that under the biofuel scenario, there is a change in seasonal P and ET. In the growing seasons (i.e., April–June), P decreases and ET increases. Evapotranspiration increases until soil moisture nears wilting point, eliminating transpiration and inhibiting further decline in soil moisture [19]. Decrease in P and increase in ET suggests the possibility for a higher magnitude of change in Q seasonally than annually, and we recommend that this possibility should be explored further with detailed hydrologic models.

This study examines landscape induced climate change and ignores projected climate change due to atmospheric concentration of greenhouse gases (GHGs). There is a strong coupling among landscape processes, atmospheric GHGs and climate, since landscape acts as a sink or source to GHGs like CO_2_ which affect the distribution of heat over land and in the atmosphere and feedback to the climate [55]. However, these processes are often uncoupled when we make future projections, and this is likely to introduce biases in the projection of future changes in climate. Thus, we recommend the direct coupling of landscape and other climate forcing factors (e.g., GHGs) to better predict the range of future climate change and its impacts on environmental resources [19]. In this study, we examine landscape induced climate change and its impact on Q on an annual basis. Changing landscape and climate alter not only Q, but also stream quality due to changes in the nutrient and sediment content of runoff, particularly from agricultural fields. Evaluation of stream quality under landscape driven climate change is thus recommended as a topic for future research.

In this study, we find interaction between land use change and climate change in Q that is not considered in previous studies. Although processes related to landscape characteristics, such as soil moisture, infiltration, and surface roughness could affect Q, but are not considered in estimation in Q in this study, we assume that the effect on streamflow of changing climate is larger than that resulting from change in landscape characteristics [56]–[58]. For example, Tu [56] examined change in streamflow in eastern Massachusetts under three different climate change scenarios under IPCC and land use scenarios relative to current conditions, individually and in combination. Tu [56] found that the change in streamflow under both climate and land use scenarios is similar to the streamflow changes examined under a climate change scenario only. Although the process-based hydrologic models (e.g., Soil and Water Assessment Tool (SWAT) and the Variable Infiltration Capacity (VIC)) have the ability to integrate the effects of land use change and climate change on Q and streamflow quality, they are computationally expensive given that they operate at the field- or sub-field scale and need to be calibrated with large amounts of empirical data. Using the method developed here, areas of concern can be identified and then detailed hydrologic models can be used to examine in more detail the specific trade-offs between land use and management options and streamflow.

The results here suggest that changes induced in the climate system by biofuel crop production may increase stress on the water resources of the High Plains. Under the biofuel scenario, increased ET reduces soil moisture [19], and lower soil moisture during the growing season can cause plant water stress and reduce crop yield. Irrigation can reduce water stress but additional irrigation will increase pressure on already strained water resources in arid agricultural regions such as the High Plains. The predicted 20% decline in Q in the biofuel crop producing region under the biofuel scenario would exacerbate on-going conflicts over water allocation between agriculture and other uses. As we develop and implement policies to pursue more sustainable cellulosic biofuel production, we should carefully consider potential water limitations and other impacts to the hydrologic cycle.

## Supporting Information

Figure S1 Location of stream gauges used in this study and major water resource regions in the U.S.
(TIF)

Figure S2 Relationship between climate elasticity of streamflow and aridity index in the U.S. watersheds. Precipitation elasticity of streamflow versus aridity index (left); Evapotranspiration elasticity of streamflow versus aridity index (right).
(TIF)

Figure S3 Hydro-climatology of the conterminous US; (A) Aridity Index (Ø); (B) Runoff Coefficient; (C) Correlation coefficient between precipitation and streamflow; and (D) Dominant land cover of the watersheds in year 2006.
(TIF)

Figure S4Standard deviation of (A) ε_p_; (B) ε_pet_; (C) mean annual change (%) in precipitation; and (D) mean annual change (%) in PET under the biofuel scenario relative to baseline scenarios. Standard deviation of elasticity estimates here reflects the variability in elasticity estimates among seven non-parametric approaches from their mean estimate, computed as 
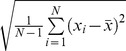
 where N = 7, 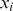 is ε_pet(i)_ or ε_p(i)_ where i represents a non-parametric approach and 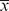 is the mean of ε_pet (i)_ or ε_p (i)_ from seven non-parametric approaches for a given location. Standard deviation of precipitation and PET change under the biofuel scenario here reflects the inter-annual variability in changes in P or PET, and is computed as 
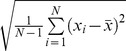
 where N = 24, the number of years between 1981 and 2004, x_i_ is 
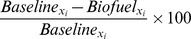
 where Baseline_xi_ and Biofuel_xi_ represent P or PET in a year i in a given location under the baseline and biofuel scenarios, respectively, and 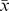 is the mean of x_i_.(TIF)

Figure S5 Change in mean annual runoff over 1981–2004 expressed in A) percentage change; B) absolute change (millimeters).
(TIF)

Figure S6 Change in streamflow volume when mean annual P and PET are varied by (A) adding and (B) subtracting a standard deviation of P and PET; (C) adding a standard deviation of elasticity (ε_p_ and ε_pet_) estimates; and (D) subtracting a standard deviation of elasticity estimates among seven non-parametric approaches.
(TIF)

Figure S7 Streamflow prediction when mean annual P and PET are varied by (A) adding, and (B) subtracting a standard deviation of P holding PET constant at its mean annual value.
(TIF)

Table S1 Values of land use parameters in the NOAH land surface model as coupled within the WRF regional climate model. Text in bold face are the new land use categories and the associated parameterization of these categories used in the switchgrass scenario. Meanings of the parameters are listed below the table (from Anderson et al. [s19]).
(DOCX)

Table S2 Mathematical expression of f(∅) and f’(∅) across the studies.
(DOCX)

Text S1 Climate Elasticity of Streamflow.
(DOCX)
