# Supplementary figures and images for: Streamflow Impacts of Biofuel Policy-Driven Landscape Change
Source: PLoS One. 2014 Oct 7;9(10):e109129. doi: 10.1371/journal.pone.0109129 (PMC4188602; doi:10.1371/journal.pone.0109129)

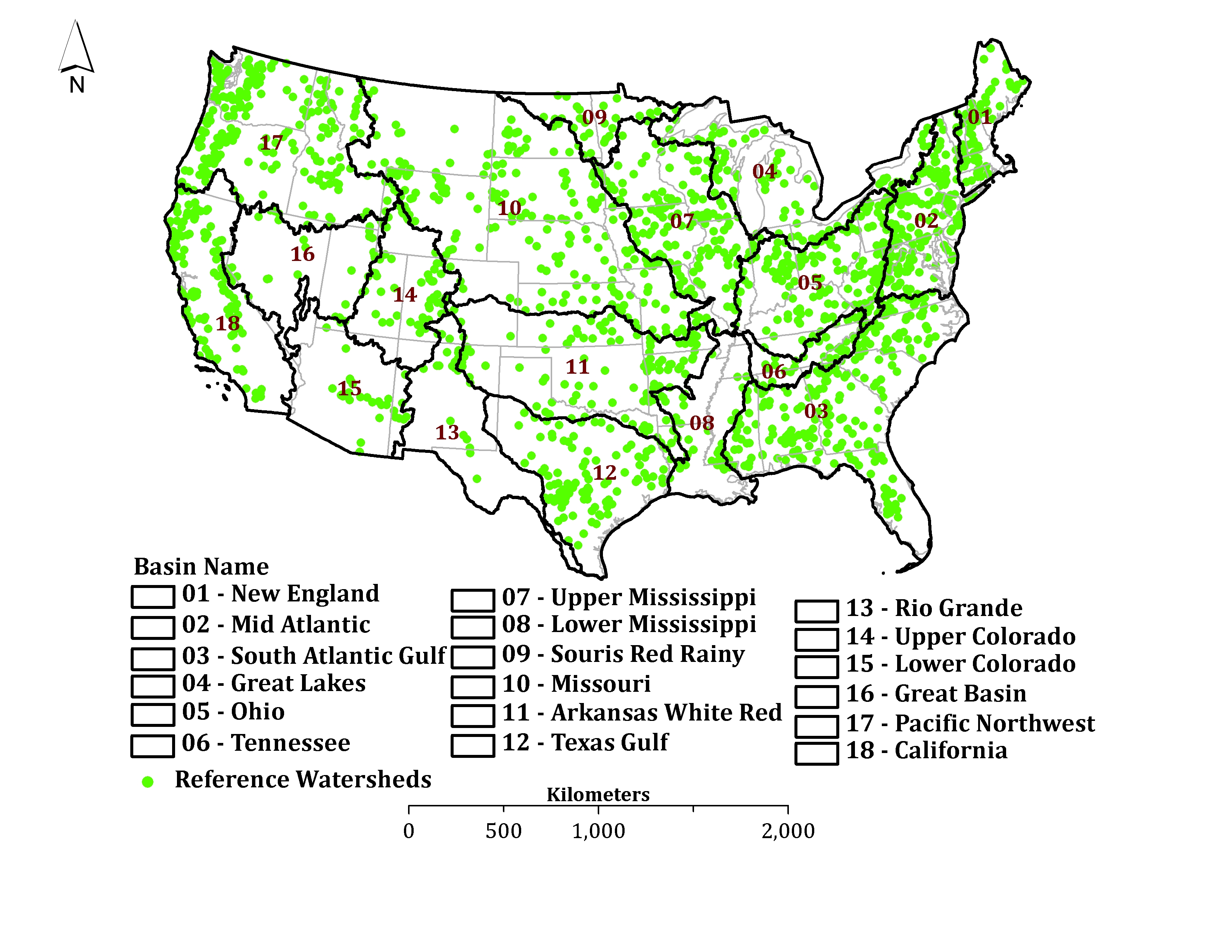

Supplement: Figure S1 — Location of stream gauges used in this study and major water resource regions in the U.S. (TIF) [file pone.0109129.s001.tif]

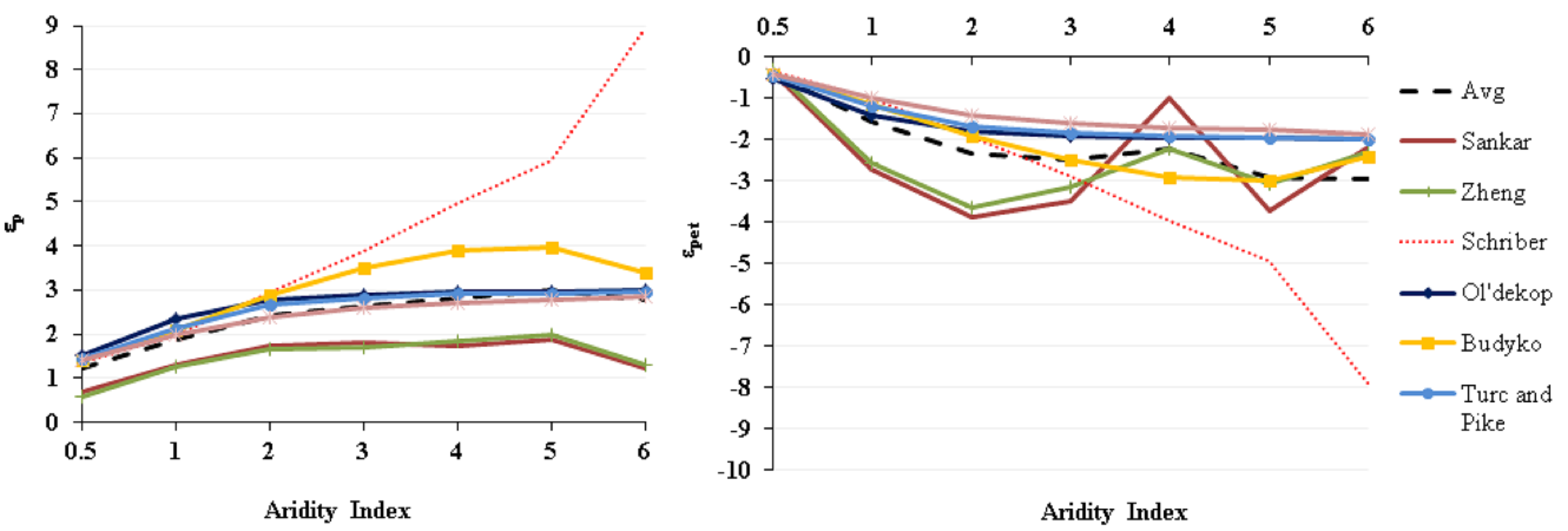

Supplement: Figure S2 — Relationship between climate elasticity of streamflow and aridity index in the U.S. watersheds. Precipitation elasticity of streamflow versus aridity index (left); Evapotranspiration elasticity of streamflow versus aridity index (right). (TIF) [file pone.0109129.s002.tif]

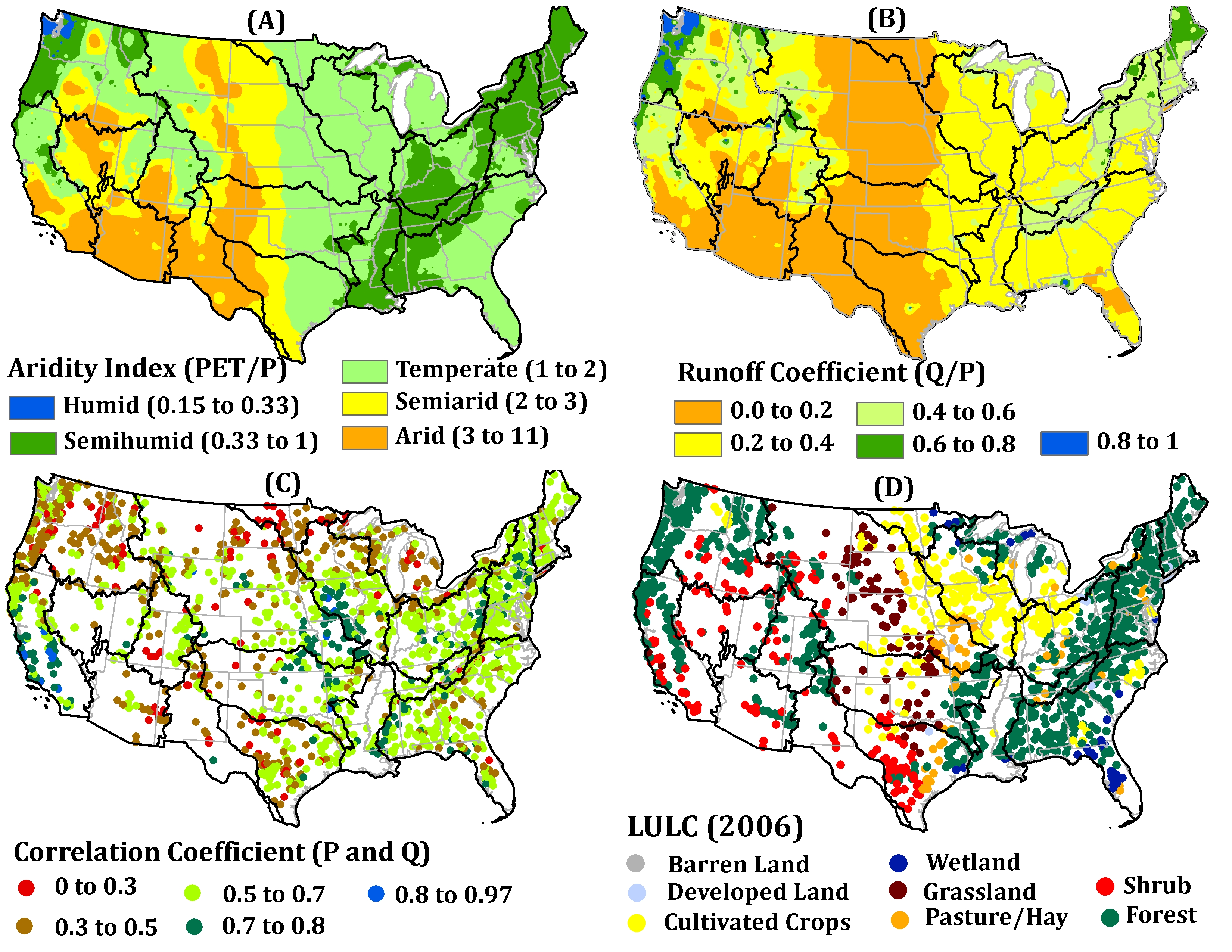

Supplement: Figure S3 — Hydro-climatology of the conterminous US; (A) Aridity Index (Ø); (B) Runoff Coefficient; (C) Correlation coefficient between precipitation and streamflow; and (D) Dominant land cover of the watersheds in year 2006. (TIF) [file pone.0109129.s003.tif]

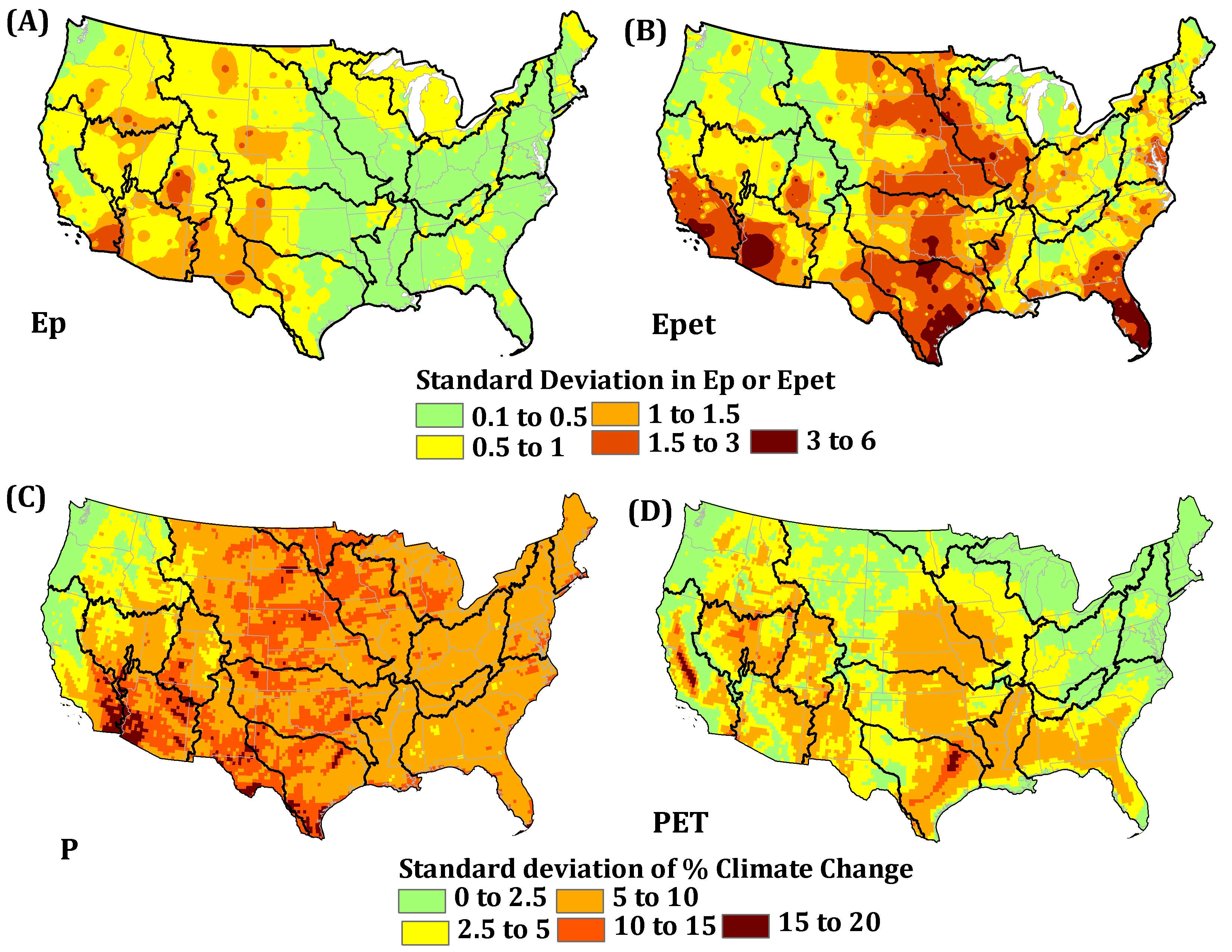

Supplement: Figure S4 — Standard deviation of (A) εp; (B) εpet; (C) mean annual change (%) in precipitation; and (D) mean annual change (%) in PET under the biofuel scenario relative to baseline scenarios. Standard deviation of elasticity estimates here reflects the variability in elasticity estimates among seven non-parametric approaches from their mean estimate, computed as where N = 7, is εpet(i) or εp(i) where i represents a non-parametric approach and is the mean of εpet (i) or εp (i) from seven non-parametric approaches for a given location. Standard deviation of precipitation and PET change under the biofuel scenario here reflects the inter-annual variability in changes in P or PET, and is computed as where N = 24, the number of years between 1981 and 2004, xi is where Baselinexi and Biofuelxi represent P or PET in a year i in a given location under the baseline and biofuel scenarios, respectively, and is the mean of xi. (TIF) [file pone.0109129.s004.tif]

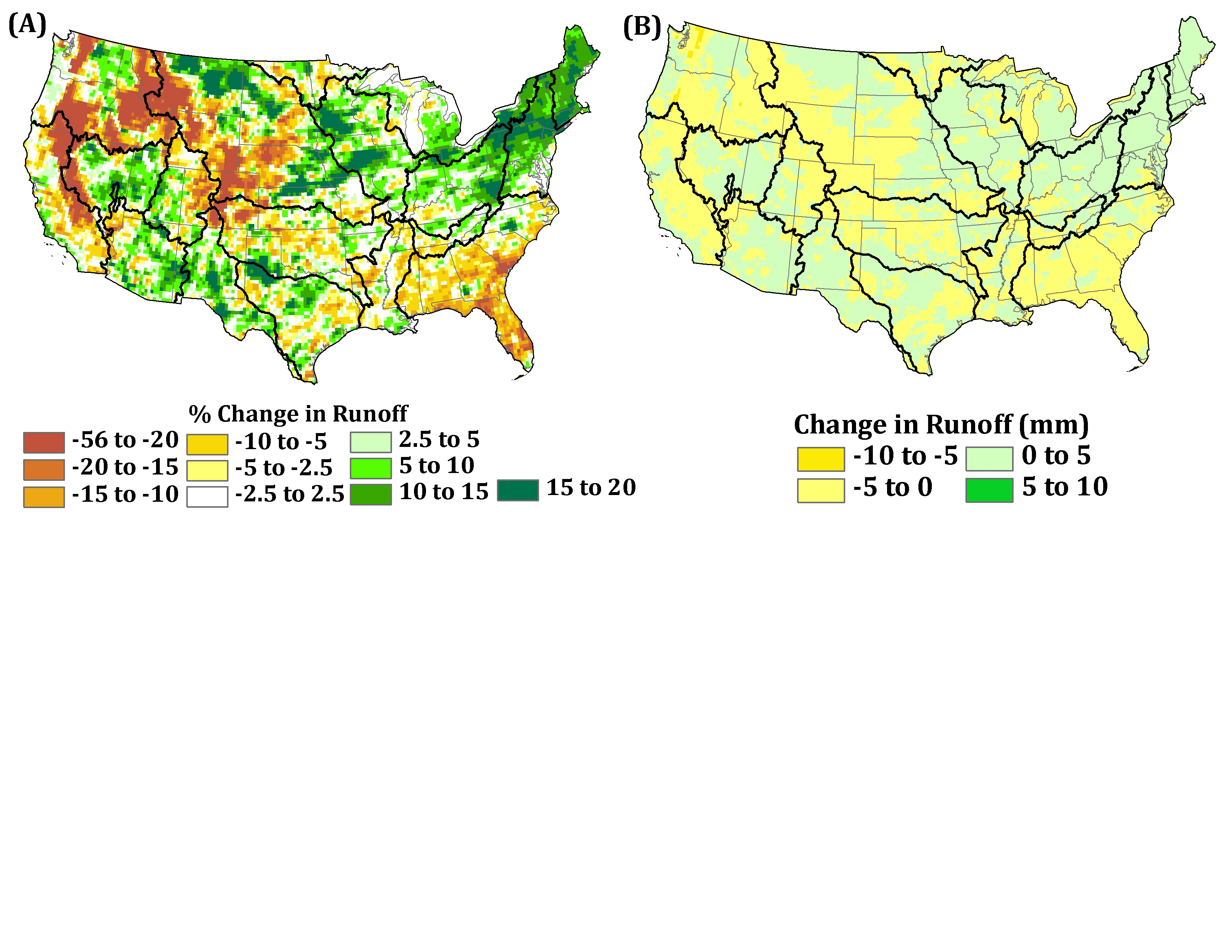

Supplement: Figure S5 — Change in mean annual runoff over 1981–2004 expressed in A) percentage change; B) absolute change (millimeters). (TIF) [file pone.0109129.s005.tif]

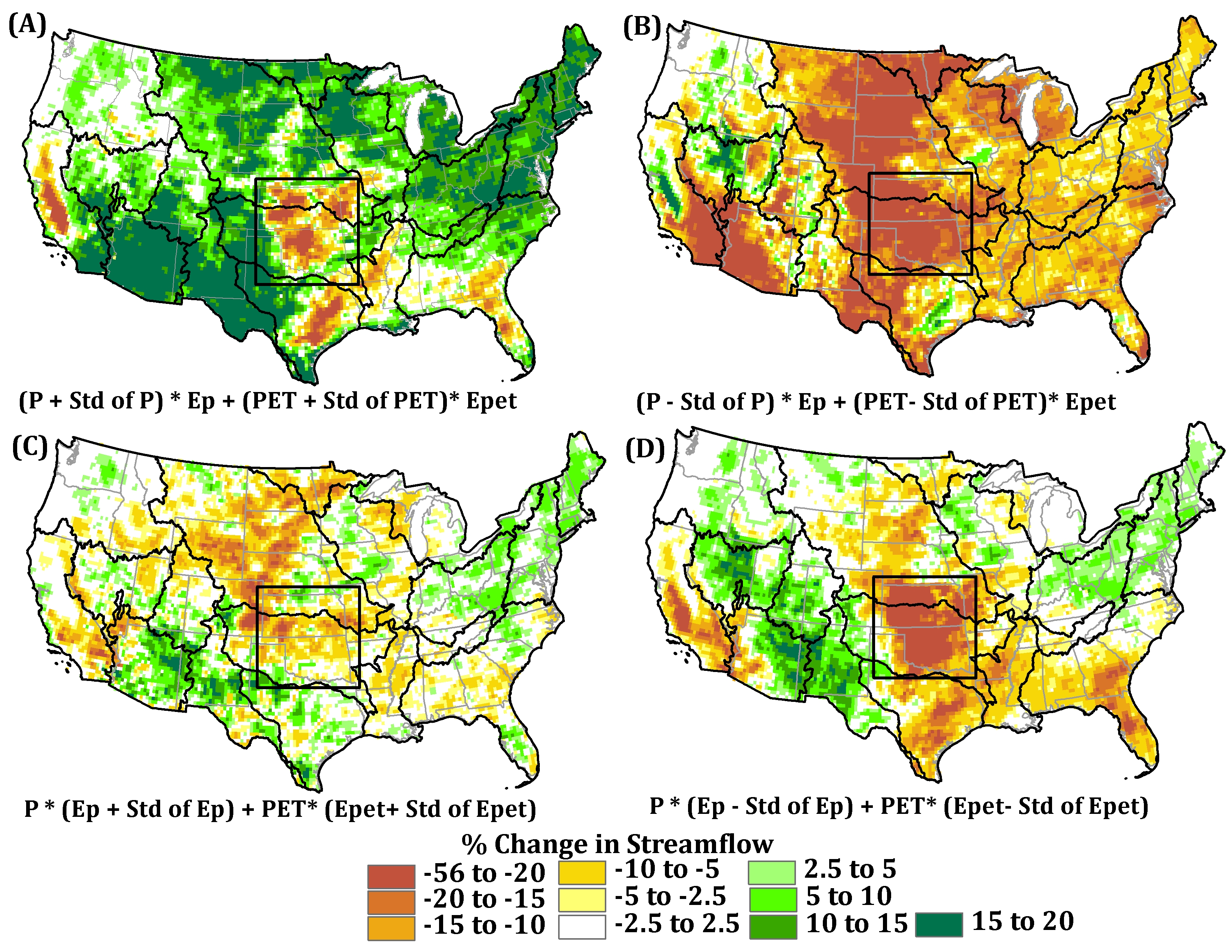

Supplement: Figure S6 — Change in streamflow volume when mean annual P and PET are varied by (A) adding and (B) subtracting a standard deviation of P and PET; (C) adding a standard deviation of elasticity (εp and εpet) estimates; and (D) subtracting a standard deviation of elasticity estimates among seven non-parametric approaches. (TIF) [file pone.0109129.s006.tif]

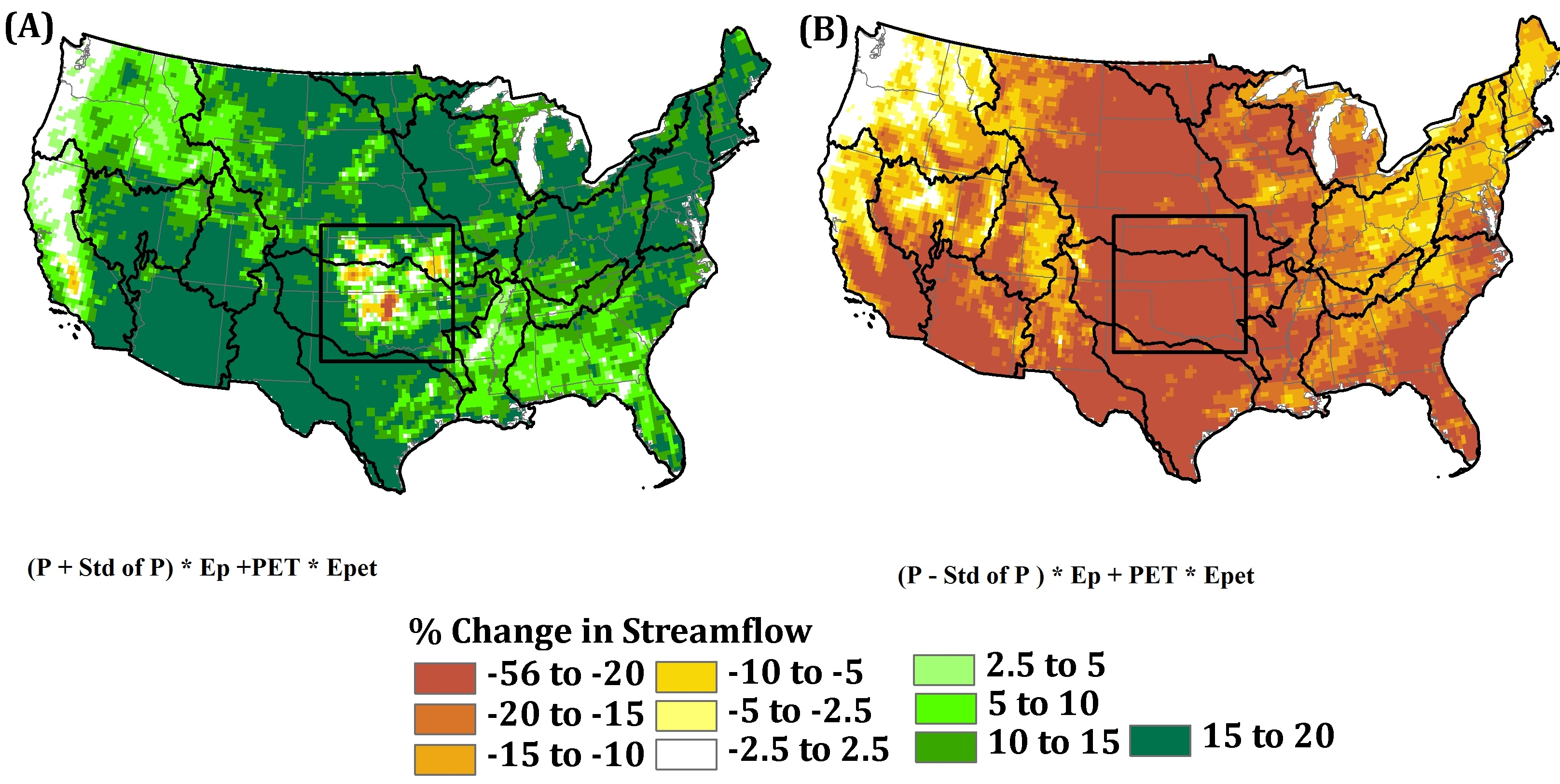

Supplement: Figure S7 — Streamflow prediction when mean annual P and PET are varied by (A) adding, and (B) subtracting a standard deviation of P holding PET constant at its mean annual value. (TIF) [file pone.0109129.s007.tif]
