# Supplementary material for: Streamflow Impacts of Biofuel Policy-Driven Landscape Change
Source: PLoS One. 2014 Oct 7;9(10):e109129. doi: 10.1371/journal.pone.0109129 (PMC4188602; doi:10.1371/journal.pone.0109129)
Supplement: Table S1 — Values of land use parameters in the NOAH land surface model as coupled within the WRF regional climate model. Text in bold face are the new land use categories and the associated parameterization of these categories used in the switchgrass scenario. Meanings of the parameters are listed below the table (from Anderson et al. [s19]). (DOCX) [file pone.0109129.s008.docx]

Table S1: Values of land use parameters in the NOAH land surface model as coupled within the WRF regional climate model. Text in bold face are the new land use categories and the associated parameterization of these categories used in the switchgrass scenario. Meanings of the parameters are listed below the table (from Anderson et al. [s19]).

| **Type** | **a_max_** | **a_min_** | **z_0max_** | **z_0min_** | **sh** | **Rt** | **rs** | **rgl** | **hs** | **snup** | **LAI_max_** | **LAI_min_** |
| --- | --- | --- | --- | --- | --- | --- | --- | --- | --- | --- | --- | --- |
| Dryland crop | 0.23 | 0.17 | 0.15 | 0.05 | 0.80 | 3 | 40 | 100 | 36.25 | 0.04 | 5.68 | 1.56 |
| Irrigated crop | 0.25 | 0.20 | 0.10 | 0.02 | 0.80 | 3 | 40 | 100 | 36.25 | 0.04 | 5.68 | 1.56 |
| Mixed dryland/irrigated | 0.23 | 0.18 | 0.15 | 0.05 | 0.80 | 3 | 40 | 100 | 36.25 | 0.04 | 4.50 | 1.00 |
| Crop/Grassland | 0.23 | 0.18 | 0.14 | 0.05 | 0.80 | 3 | 40 | 100 | 36.25 | 0.04 | 4.29 | 2.29 |
| Crop/Woodland | 0.20 | 0.16 | 0.20 | 0.20 | 0.80 | 3 | 70 | 65 | 44.14 | 0.04 | 4.00 | 2.00 |
| Grassland | 0.20 | 0.16 | 0.12 | 0.10 | 0.80 | 3 | 40 | 100 | 36.35 | 0.04 | 0.52 | 2.90 |
| **Switchgrass /Grassland** | **0.23** | **0.18** | **0.13** | **0.08** | **0.80** | **3** | **40** | **100** | **36.30** | **0.04** | **4.91** | **0.89** |
| **Switchgrass /Cropland** | **0.23** | **0.17** | **0.13** | **0.08** | **0.80** | **3** | **40** | **100** | **36.25** | **0.04** | **6.38** | **1.72** |
| **Grassland /Switchgrass** | **0.23** | **0.18** | **0.12** | **0.09** | **0.80** | **3** | **40** | **100** | **36.25** | **0.04** | **3.98** | **0.71** |
| **Cropland /Grassland** | **0.23** | **0.17** | **0.12** | **0.09** | **0.80** | **3** | **40** | **100** | **36.25** | **0.04** | **5.92** | **1.72** |

a_min_ = minimum albedo (dimensionless)

a_max_ = maximum albedo (dimensionless)

z_0max_ = maximum aerodynamic roughness length (m)

z_0min_ = maximum aerodynamic roughness length (m)

sh = maximum areal fraction of green vegetation (dimensionless)

rt = number of root layers (dimensionless)

rs = minimum canopy resistance (s m^-1^)

rgl = sensitivity factor of canopy resistance due to solar radiation

hs = sensitivity factor of canopy resistance due to vapor pressure deficit

snup = threshold snow water equivalent for 100% snow cover (m)

LAI_max_ = maximum leaf area index (dimensionless)

LAI_min_ = minimum leaf area index (dimensionless
