# Supplementary material for: Streamflow Impacts of Biofuel Policy-Driven Landscape Change
Source: PLoS One. 2014 Oct 7;9(10):e109129. doi: 10.1371/journal.pone.0109129 (PMC4188602; doi:10.1371/journal.pone.0109129)
Supplement: Table S2 — Mathematical expression of f(∅) and f’(∅) across the studies. (DOCX) [file pone.0109129.s009.docx]

Table S2: Mathematical expression of $f\left( \emptyset\right)$and $f'\left( \emptyset\right)$ across the studies

| Studies | f(∅) | f’(∅) |
| --- | --- | --- |
| Schreiber (1904) | $1-e^{-\emptyset}$ | $e^{-\emptyset}$ |
| Ol’dekop (1911) | $\emptyset tanh\left(1/\emptyset\right)$ | $tanh\left(1/\emptyset\right)-4/\left[\emptyset\left(e^{-1/\emptyset}+e^{1/\emptyset}\right)^{2}\right]$ |
| Budyko (1948) | $\left[\emptyset tanh\left(1/\emptyset\right)\left(1-e^{-\emptyset}\right)\right]^{0.5}$ | $\begin{matrix} 1/2\left[\emptyset tanh\left(1/\emptyset\right)\left(1-e^{-\emptyset}\right)\right]^{-0.5} \\ \left[\left(tanh\left(1/\emptyset\right)-1/\emptyset sech^{2}\left(1/\emptyset\right)\right)\left(1-e^{-\emptyset}\right)+\emptyset tanh\left(1/\emptyset\right)e^{-\emptyset}\right] \end{matrix}$ |
| Turc (1984) and Pike (1964) | $1/\sqrt{(1+\emptyset^{-2})}$ | $1/\emptyset^{3}\left[1+\left(1/\emptyset\right)^{2}\right]^{1.5}$ |
| Zhang *et al.*(2000)^*^ | $\left(1+w\emptyset\right)/\left(1+w\emptyset+1/\emptyset\right)$ | $\left[w+2w/\emptyset-1+1/\emptyset^{2}\right]/\left[1+w\emptyset+1/\emptyset\right]^{2}$ |

*The variable w is set to 1.
