# Supplementary material for: Streamflow Impacts of Biofuel Policy-Driven Landscape Change
Source: PLoS One. 2014 Oct 7;9(10):e109129. doi: 10.1371/journal.pone.0109129 (PMC4188602; doi:10.1371/journal.pone.0109129)
Supplement: Text S1 — Climate Elasticity of Streamflow. (DOCX) [file pone.0109129.s010.docx]

## Climate elasticity of streamflow

Sankarasubramanian *et al* [S1] proposed a robust non-parametric estimator to estimate $\varepsilon_{x}$via Monte Carlo experiment expressed as:

$\text{ε}_{\text{X}}=\text{median}\left(\frac{\text{Q}_{\text{t}}-\text{ }\bar{\text{Q}}}{\text{X}_{\text{t}}-\text{ }\bar{\text{X}}}\text{ }\frac{\bar{\text{X}}}{\bar{\text{Q}}}\text{ }\right)$ (s1)

A value of $\left(\frac{\text{Q}_{\text{t}}-\text{ }\bar{\text{Q}}}{\text{X}_{\text{t}}-\text{ }\bar{\text{X}}}\text{ }\frac{\bar{\text{X}}}{\bar{\text{Q}}}\text{ }\right)$ is calculated for a watershed using a pairs of X_t_ and Q_t_ (i.e., P and Q for ε_p_, PET and Q for ε_pet_) in annual time series. This approach has been compared with detailed hydrologic models and is shown to be as or more robust than complex watershed models for evaluating the sensitivity of streamflow to climate [S1-S3]. It has also been suggested that the non-parametric estimates may be quite useful for validating hydro-climatologic investigations [S1]. However, equation s1 is statistically weak when the annual time series of climate information of a watershed is short. To overcome the weakness associated with short time series, Zheng *et al* [S4] proposed an alternative non-parametric estimator of ε_x_:

$\text{ε}_{\text{x}}=\text{ρ}_{\text{X},\text{Q}}\left(\frac{\text{C}_{\text{Q}}}{\text{C}_{\text{X}}}\right)$ (s2)

where $\text{ρ}_{\text{X},\text{Q}}$is the correlation coefficient (i.e., correlation between X and Q), and C_Q_ and C_x_ are the coefficients of variation of Q and X, respectively. This form of non-parametric estimator indicates that higher are $\text{ρ}_{\text{X},\text{Q}}$and $\frac{\text{C}_{\text{Q}}}{\text{C}_{\text{X}}}$the more sensitive the streamflow is to the climate changes. Studies have extended the use of the aridity index (Ø) to obtain an estimate of change in annual runoff due to change in climate [s5-s10]. Aridity index is defined as the ratio of PET and P [S1, S4, S11]. Mean annual runoff over a long period of time is estimated using P and PET as:

$\bar{\text{Q}}=\bar{\text{P}}-\bar{\text{E}}_{\text{a}}$ (s3)

E_a =_ P f(Ø) (s4)

Where $\bar{\text{Q}}$, $\bar{\text{P}}$and $\bar{\text{E}}_{\text{a}}$are mean annual Q, P and actual evapotranspiration. Various functional forms of Ø, i.e., f (Ø) are used to derive E_a_ as well as runoff [S11]. Arora [S11] describes the multiple forms of f (Ø) and f’ (Ø) used in prior studies (Table S2) and derives a relationship to estimate $\varepsilon_{P}$ as:

$$\varepsilon_{P}=1+\frac{\emptyset\text{f' (}\emptyset)}{1-f(\emptyset)'}$$

And $\varepsilon_{P}+ \varepsilon_{pet}=1$ (5)

We use ε_x_ measures that are invariant between the baseline and biofuel scenarios to compute changes in Q in the biofuel scenario. Changing climate in the biofuel scenario influences plant productivity which might change infiltration, storage capacity of the soil and the streamflow of a watershed. However, the change in infiltration and storage capacity of a watershed due to changes in cropping system over the 24 year period are smaller than the annual change in precipitation, evapotranspiration and runoff fluxes [S12]. Thus invariant infiltration and storage capacity should cause little if any differences in ε_x_ between the baseline and biofuel scenarios. Historic mean annual Q, P and PET with equations s1, s2 and s5 are used to compute ε_x_ in 1,845 watersheds across the conterminous U.S. Various forms of f (Ø) and f’ (Ø) used in prior studies (Table S2) are used to compute ε_x_.

Precipitation elasticity of streamflow increases with increasing Ø regardless of differences in the approaches used to compute elasticity estimates, whether they include Ø [S5-S10] or exclude it [S1,S4] (Figure S2). All approaches show an increase in ε_p_ until Ø reaches 2. Except for the Schreiber and Budyko methods, ε_p_ reaches a plateau for Ø larger than 2. When PET exceeds P in arid regions, actual evapotranspiration approaches P, and ε_p_ remains unchanged with increasing Q. Although Sankarasubramanian *et al* [s1] and Zheng *et al* [s4] use historic P and Q information but not Ø, ε_p_ estimated using their approaches follow a trend similar to the other models; however estimated values are lower.

## Data

***Watershed and streamflow database***

The geographic location and watershed boundaries are obtained from the GAGESII database maintained by the U.S. Geological Survey (USGS). This database represents 9,068 watersheds in the conterminous U.S. Watersheds that are either identified as reference or are the part of Hydro-Climatic-Data Network (HCDN) are used. Reference watersheds in the GAGESII [S13] and watersheds in the HCDN database [S14] are watersheds that are not subject to regulation or diversion. Details of these datasets are discussed in Falcone *et al* [S14] and Slack *et al* [S14]. Reference and HCDN watersheds that have 20 plus years of streamflow record between 1950 and 2009, and have basin areas greater than 50 km^2^are considered. The size of the smallest watershed is larger than the size of a pixel of raster data (i.e., 4 km x 4 km) based on which historic climate information is collected. This provides a total of 1,845 watersheds. Annual streamflow records for these watersheds are obtained from the National Water Information System [S15] website. Streamflow data in cubic feet per second are converted into millimeters per year based on watershed size. Watersheds used in the study are shown in Figure S1.

***Climate database***

A time series of annual P and PET from 1950-2009 in each watershed is derived from raster grids available from the PRISM website [S16] and watershed boundary information using the ArcGIS function “Zonal Statistics” [S17]. These climate data are then combined with the streamflow records of the same period to compute $\varepsilon_{x}$ of the watersheds. The PRISM website provides a series of monthly and annual temperature and P grids at 4 km x 4 km resolution for the conterminous U.S. Monthly PET for the conterminous U.S. is computed using the method introduced by Hargreaves and Samani [S18] which requires monthly average minimum and maximum temperature and extraterrestrial solar radiation (equation s6). Extraterrestrial solar radiation is estimated by computing solar radiation over a 0.1°grid using the method described in Allen *et al* [s19].

$\text{PET}=0.0135\times\left(\text{KT}\right)\times\left(\text{R}_{\text{a}}\right)\times(\text{TD})^{0.5}\times\left(\text{TC}+17.8\right)$ (s6)

where PET is the potential evapotranspiration (mm d^-1^), TD = T_max_ – T_min_ (°C), and TC is the average daily temperature (°C), Ra is extraterrestrial radiation (mm/day) and KT is empirical coefficient expressed as follows:

$\text{KT}=0.00185\times\left(\text{TD}\right)^{2}-0.0433\times\left(\text{TD}\right)+0.4023$ (s7)

We do not use Penman Monteith or similar methods to estimate PET because only few meteorological stations observe all of the required parameters (i.e., air temperature, wind speed, relative humidity, and solar radiation). The number of stations where reliable data for these parameters exist is even smaller [S20]. The method of Hargreaves and Samani has lower data requirements and is a method that is preferred over other temperature-based methods [S1, S21].

## Change in streamflow under the biofuel scenario

Climate elasticity of streamflow measures (i.e., ε_p_ and ε_pet_ ) for the conterminous U.S. are computed by interpolating the elasticity values computed for the reference watersheds using the Inverse Distance Weighting (IDW) function in ArcGIS [S17]. For interpolation, the “variable search radius” option in the IDW function is used. The residuals between the interpolated and observed value for precipitation and evapotranspiration elasticity at the observation points are found to be very minimal (i.e., RMSE<0.0005 for precipitation and RMSE<0.006 for evapotranspiration).

## Hydro-climatology of the conterminous U.S

The geographical distribution of Ø, elasticity estimates, and LULC indicate that streamflow in arid and semi-arid regions is highly sensitive to changes in P and PET; streamflow is less correlated with P in these regions which are dominated by grass, shrubs and pasture. The geographic distribution of Ø, runoff coefficient, a Pearson’s correlation coefficient between P and Q, and dominant land use cover in the watersheds considered in this study are provided in the supporting materials (Figures S3). Also, arid regions tend to have a lower runoff ratio. Over a large part of the eastern and far western U.S., a decrease in Ø is associated with combination of multiple factors, including increased runoff ratio, increased dominance of the forested land, and the larger correlation between Q and P. Semiarid and arid regions receive nearly as much P as they lose via ET and thus there is usually a lower runoff in these regions. Soils in these regions have lower water holding capacity, and are less efficient in buffering runoff during high P events, thus, increasing Q. This leads to higher $\varepsilon_{p}$ in arid regions. In contrast, humid and semi-humid regions have nearly as much Q as P. Soils in these regions have higher water holding capacity, providing more buffering capacity during extreme P events and thus have lower ε_p_. Difference in timing of snow melt which contributes to streamflow in the western U.S. usually results in a higher runoff coefficient there.

The Q, P and PET observed in a region are closely related to the landscape characteristics of that region. That is, elasticity estimates implicitly account for landscape effects including associated moisture and energy balances. For example, Q in forested regions is less sensitive to changes in P and ET than Q in grassland and cropland regions (Figure S3). Thus cropland and grassland are of particular interest as we seek to understand the impact of climate change on Q.

## References

S1. Sankarasubramanian A, Vogel RM, Limbrunner JF (2001) Climate elasticity of streamflow in the United States. Water Resources Research 37: 1771-1781.

S2. Chiew F, Teng J, Vaze J, Kirono D (2009) Influence of global climate model selection on runoff impact assessment. Journal of Hydrology 379: 172-180.

S3. Chiew FHS (2006) Estimation of rainfall elasticity of streamflow in Australia. Hydrological Sciences 51: 613-625.

S4. Zheng H, Zhang L, Zhu R, Liu C, Sato Y, Fukushima Y (2009) Responses of streamflow to climate and land surface change in the headwaters of the Yellow River Basin. Water Resources Research 45 W00A19.

S5. Zhang L, Dawes W, Walker G (2001) Response of mean annual evapotranspiration to vegetation changes at catchment scale. Water Resources Research 37: 701-708.

S6. Pike J (1964) The estimation of annual run-off from meteorological data in a tropical climate. Journal of Hydrology 2: 116-123.

S7. Budyko MI (1963) Evaporation under natural conditions, Israel Program for Scientific Translations; [available from the Office of Technical Services, US Dept. of Commerce, Washington].

S7. Turc L (1954) The water balance of soils. Relation between precipitation evaporation and flow. Ann Agron. 5: 491-569.

S8. Ol’Dekop E (1911) On evaporation from the surface of river basins. Trans. Met. Obs. lurevskogo, Univ. Tartu, 4.

S9. Schreiber P (1904) Über die Beziehungen zwischen dem Niederschlag und der Wasserführung der Flüsse in Mitteleuropa. Meteorologische Zeitschrift 21: 441-452.

S10. Arora VK (2002) The use of the aridity index to assess climate change effect on annual runoff. Journal of Hydrology 265: 164-177.

S11. Koster RD, Suarez MJ (1999) A simple framework for examining the interannual variability of land surface moisture fluxes. J Clim 12: 1911-1917.

S12. Falcone JA, Carlisle DM, Wolock DM, Meador MR (2010) GAGES: A stream gage database for evaluating natural and altered flow conditions in the conterminous United States. Ecology 91: 621-621.

S13. Slack JR, Landwehr JM (1994) Hydro-climatic data network (HCDN): A US Geological Survey streamflow data set for the United States for the study of climate variations. US Geological Survey: 1874-1988.

S14. NWIS – National Water Information System. USGS surface-water data for the nation. http://waterdata.usgs.gov/usa/nwis/sw (accessed July 18, 2011).

S15. PRISM – PRISM Climate Group. http://www.prism.oregonstate.edu (accessed July 17, 2011).

S16. ESRI. [Online]. Web help. http://webhelp.esri.com/arcgisdesktop/9.2/index.cfm? (accessed April 6,2012).

S17. Hargreaves GH, Samani ZA (1982) Estimating potential evapotranspiration. Journal of the Irrigation and Drainage Division 108: 225-230.

S18. Allen RG, Pereira LS, Raes D, Smith M (1998) Crop evapotranspiration-guidelines for computing crop water requirements-FAO irrigation and drainage paper, 56. FAO, Rome 300, 6541.

S19. Droogers P, Allen RG (2002) Estimating reference evapotranspiration under inaccurate data conditions. Irrig Drain Syst 16: 33-45.
